# Evolutionary dynamics of U12-type spliceosomal introns

**DOI:** 10.1186/1471-2148-10-47

**Published:** 2010-02-17

**Authors:** Chiao-Feng Lin, Stephen M Mount, Artur Jarmołowski, Wojciech Makałowski

**Affiliations:** 1 Institute of Bioinformatics, University of Muenster, Muenster, Germany; 2 1413 Blockley Hall, Center for Bioinformatics, 423 Guardian Dr, University of Pennsylvania, Philadelphia, PA, 19104, USA; 3 Department of Cell Biology and Molecular Genetics, University of Maryland, College Park, Maryland, 20742, USA; 4 Center for Bioinformatics and Computational Biology, University of Maryland, College Park, Maryland, 20742, USA; 5 Department of Gene Expression, Institute of Molecular Biology and Biotechnology, Adam Mickiewicz University, Poznañ, Poland

## Abstract

**Background:**

Many multicellular eukaryotes have two types of spliceosomes for the removal of introns from messenger RNA precursors. The major (U2) spliceosome processes the vast majority of introns, referred to as U2-type introns, while the minor (U12) spliceosome removes a small fraction (less than 0.5%) of introns, referred to as U12-type introns. U12-type introns have distinct sequence elements and usually occur together in genes with U2-type introns. A phylogenetic distribution of U12-type introns shows that the minor splicing pathway appeared very early in eukaryotic evolution and has been lost repeatedly.

**Results:**

We have investigated the evolution of U12-type introns among eighteen metazoan genomes by analyzing orthologous U12-type intron clusters. Examination of gain, loss, and type switching shows that intron type is remarkably conserved among vertebrates. Among 180 intron clusters, only eight show intron loss in any vertebrate species and only five show conversion between the U12 and the U2-type. Although there are only nineteen U12-type introns in *Drosophila melanogaster*, we found one case of U2 to U12-type conversion, apparently mediated by the activation of cryptic U12 splice sites early in the dipteran lineage. Overall, loss of U12-type introns is more common than conversion to U2-type and the U12 to U2 conversion occurs more frequently among introns of the GT-AG subtype than among introns of the AT-AC subtype. We also found support for natural U12-type introns with non-canonical terminal dinucleotides (CT-AC, GG-AG, and GA-AG) that have not been previously reported.

**Conclusions:**

Although complete loss of the U12-type spliceosome has occurred repeatedly, U12 introns are extremely stable in some taxa, including eutheria. Loss of U12 introns or the genes containing them is more common than conversion to the U2-type. The degeneracy of U12-type terminal dinucleotides among natural U12-type introns is higher than previously thought.

## Background

In most eukaryotic protein-coding genes, the sequences found in mature messenger RNAs (exons) are discontinuous, and are separated by intervening sequences known as introns. The removal of introns, in a process known as pre-mRNA splicing, is performed by the spliceosome, which consists of the U1, U2, U4, U5 and U6 snRNPs (small nuclear ribonucleoproteins), and more than 200 non-snRNP proteins [1-3].

The terminal dinucleotides GT and AG at the 5' and 3' end of introns, respectively, are nearly universal [4-6]. However, compilation of introns with non-consensus splice sites [7,8] led to the discovery of a minute class of introns. These introns contained an extended and nearly invariant 5' splice site (ATATCCTT at +1 to +8 positions starting from the 5' junction), a more pronounced Branch Point Site (BPS, TCCTTAAC), and an AC acceptor site. Possible base-pairing between the 5' splice site and U11 snRNA, and between the BPS and U12 snRNA, prompted Hall and Padgett [8] to propose that the low-abundance U11 and U12 snRNAs are involved in the splicing of this new class of introns. Biochemical analysis of the splicing of one such intron [9,10] led to the discovery of a distinct spliceosome containing U11, U12, U5 and two new snRNAs (U4atac and U6atac). Later, a set of novel proteins specific to the minor class of snRNPs, but generally homologous to proteins in the major class of snRNPs, was described [11].

Introns in the minor class were originally called AT-AC introns for their unusual terminal dinucleotides. However, Dietrich *et al. *[12] demonstrated that an AT-AC intron with double mutations at the first and last nucleotides, which turned the termini to GT-AG, could be properly removed by a minor-type spliceosome. On the other hand, the splicing of intron 21 of the human *SCN4A *gene, which has AT-AC termini, requires a major-type spliceosome [12]. Since terminal dinucleotides do not necessarily indicate the splicing pathway that an intron undergoes, Dietrich *et al. *[12] coined the terms "U12-dependent" and "U2-dependent" to denote the introns and spliceosome involved in the minor and major splicing pathway, respectively. This nomenclature has been widely accepted and frequently a shorter version, "U12-type" and "U2-type", has been used.

In the first study that investigated the evolution of U12-type introns, Burge *et al. *[13] proposed that U12-type introns can be "converted" into U2-type introns. They described eight sets of U12-type introns having U2-type orthologs (introns occurring at an orthologous codon position and the same intron phase) as the outcome of U12-U2 conversion. Given that U12-type splicing signals are more conserved, they noted that conversion from a U12- to U2-type is more likely than conversion from a U2- to a U12-type. They also found orthologous U12-type introns that differ in subtype (GT-AG vs. AT-AC terminal dinucleotides), and referred to the change as a "subtype switch." Subtype switches provide a possible evolutionary mechanism for type switches, since as little as one additional mutation (C to G at +5 position of the 5' splice site) can convert a GT-AG U12-type intron into a GT-AG U2-type. This also suggests that a subtype switch from AT-AC to GT-AG can serve as an intermediate stage for a type conversion from U12 to U2.

The wide spread of the U12-type spliceosome in eukaryotes and its independent loss in distantly related species was recently studied by computational scanning for spliceosomal snRNA genes in 149 eukaryotic genomes [14]. The presence of U12-type introns and snRNAs in both plants and animals suggests that the U12-type splicing pathway existed in the common ancestor, and has been lost in some genomes, e.g. *C. elegans *and yeast. The question whether these two seemingly parallel splicing machineries descended from the same ancestor is intriguing but remains unsolved. Burge *et al. *[13] proposed the fission-fusion hypothesis; two splicing pathways diverged from one in the course of speciation (fission) and later merged (fusion) in one organism. They reasoned that the similar secondary structure and interaction between the two sets of spliceosomal snRNAs are unlikely to be the result of convergent evolution. However, Lynch and Richardson argued that the two splicing systems originated from two group II self-splicing introns, and that the proteins involved in splicing shaped the similarity between functionally analogous snRNAs [15]. In this model, homologous proteins in the two spliceosomes can be explained by the duplication and specialization of genes for proteins that were originally shared. Recently, Basu *at al. *analyzed protosplice sites in the human and *Arabidopsis thaliana *genomes and concluded that U2-type introns predate the U12-type [16]. This finding supports Lynch and Richardson's model, suggesting that U2-type introns first populated genomes to a certain intron density and the successive invasion of the ancestral genome by U12-type introns was restricted in scale because of limited space available for the insertion of new genes.

Since the publication of Burge and colleagues' evolutionary study of U12-type introns, many complete genomes have become available. U12-type introns have been computationally identified and characterized in several genomes, including *Homo sapiens *[17] and *Arabidopsis thaliana *[18]. Here we present an investigation to evaluate the relative contribution of various mechanisms, such as intron gain and loss and U12-U2 conversion, to the evolution of U12-type introns. Two databases provide access to U12-type introns that have been identified in multiple genomes: SpliceRack [19] and U12DB [20]. More U12-type introns, particularly those with AT-AC termini, were reported in U12DB because the database incorporated introns predicted by mapping EST data in addition to genomewide annotation. Furthermore, U12DB has a broader range of genomes (twenty eukaryotes), and incorporates orthology information (based on Ensembl and Inparanoid databases) among introns by organizing U12-type introns and their orthologous introns into intron clusters. Such a dataset is ideal for studying the evolution of U12-type introns. We retrieved the whole U12DB database and carried out manual inspection to assess the frequency of type switching, U12 intron loss and gain in sets of genomes. These are: 1) eutheria (*Homo sapiens*, *Mus musculus*, *Rattus norvegicus*, *Canis familiaris*), 2) vertebrates (with addition of *Monodelphis domestica*, *Gallus gallus*, *Fugu rubripes*, *Tetraodon nigroviridis*, *Danio rerio*), 3) chordates (with addition of *Ciona intestinalis*), 4) *Homo sapiens *vs. *Drosophila melanogaster*), and 5) *Homo sapiens *vs. *Arabidopsis thaliana*.

Our results reveal that U12-type introns are remarkably conserved among vertebrates. On the other hand, there has been a massive loss of U12-type introns in multiple invertebrate lineages, suggesting that the rate of U12-type intron loss varies significantly among taxa. Interestingly, one of only nineteen U12-type introns that were detected in the *D. melanogaster *genome appeared to be the first known case of U2 to U12-type conversion. In general, loss of U12-type introns is more common than their conversion to U2-type and U12 to U2 conversion occurs more frequently among U12 introns of the GT-AG subtype. We also found support for natural U12-type introns with non-canonical terminal dinucleotides (CT-AC, GG-AG, and GA-AG) that have not been previously reported.

## Results and Discussion

To study the evolution of U12-type introns, we investigated conservation and changes of the intron state among U12-type introns and their orthologs in twenty sequenced eukaryotic genomes: *Homo sapiens, Pan troglodytes, Macaca mulatta, Mus musculus, Rattus norvegicus, Canis familiaris, Bos taurus, Monodelphis domestica, Gallus gallus, Xenopus laevis, Fugu rubripes, Tetraodon nigroviridis, Danio rerio, Ciona intestinalis, Apis mellifera, Drosophila melanogaster, Anopheles gambiae, Caenorhabditis elegans*, and *Arabidopsis thaliana*. We utilized orthologous U12-type intron clusters and related data available in the U12DB [20]. Members of an orthologous intron cluster are introns in orthologous genes that are flanked by alignable exonic sequences. The status of each intron in each species was designated in the U12DB as U12, U2, ambiguous, or lost. Changes in intron state between orthologous introns were noted and manually curated.

Pairwise comparisons to the human genome (prior to the detailed investigation described below) as a reference provide an overview of the entire dataset (see Table 1). Humans were used as the reference species because the greatest number of U12-type introns has been identified in the human genome. In Table 1, the first column shows the number of U12-type introns that were included in the analysis from each genome. Not surprisingly, both the number of intron pairs compared and the number of U12-type introns conserved between the human and the compared genomes decrease with the phylogenetic distance. Consequently, U12-type introns are conserved in at least 90% of the cases between the human and non-human vertebrate genomes and in about 20% of the comparisons in invertebrates. The absence of an orthologous intron is much more frequent than U12 to U2 intron transition. This initial summary also shows that the number of U12-type introns identified in each genome varies in a manner that is best explained by the quality of genome assembly and the abundance of mRNA and EST data. For instance, the number of U12-type introns detected in the human genome is unmatched by that of other primates or mammals. The number of compared and conserved intron pairs in the human-mouse comparison is higher than in the human-chimp or human-macaque comparisons, an observation that is best accounted for by the fact that the mouse genome is better studied than the chimpanzee or macaque genomes. Finally, the number of intron clusters is clearly undercounted in the case of the two most phylogenetically distant genomes: *Drosophila *(see below) and *Arabidopsis *[21].

**Table 1:** Intron state and counts of introns in different species that are orthologous to 621 human U12-type introns.

Compared species	Numb. of availa-ble compa-risons	Numb. of absent introns	Numb. of U2 introns	Number of ambi-guous introns	Number of U12 introns	Proportion of U12 introns shared with the human genome	Total number of U12 introns in the genome	Proportion of conserved U12 introns in a given genome
*Pan troglodytes*	486	6	0	7	473	0.973	474	0.998
*Macaca mulatta*	452	14	1	7	430	0.951	434	0.991
*Mus musculus*	501	11	0	7	483	0.964	490	0.986
*Rattus norvegicus*	483	15	1	5	462	0.957	469	0.985
*Canis familiaris*	516	7	0	11	498	0.965	503	0.990
*Bos taurus*	469	9	2	9	449	0.957	455	0.987
*Monodelphis domestica*	440	12	1	5	422	0.959	428	0.986
*Gallus gallus*	391	13	0	9	369	0.944	373	0.989
*Fugu rubripes*	333	21	6	5	301	0.904	308	0.977
*Danio rerio*	301	11	6	10	274	0.910	282	0.972
*Ciona intestinalis*	154	83	29	1	41	0.266	113	0.363
*Apis mellifera*	161	122	6	2	31	0.193	34	0.912
*Drosophila melanogaster*	198	169	18	5	6	0.030	16	0.375
*Caenorhabditis elegans*	96	80	15	1	0	0.000	0	0.000
*Arabidopsis thaliana*	2	0	0	0	2	1.000	216	0.009

Results shown were automatically inferred based on information in the U12db [18]. For some species manual adjustment resulted in a different final count of U12 introns.

In addition to possible inaccuracy in genome assembly or annotation, the way U12DB was constructed also poses the following limitations for the present study. 1) The number of reported U12-type introns for non-reference genomes may be underestimated, since these genomes have not been fully searched. 2) Identification of the orthologous intron relies on correct assignment of orthologous genes since only sequences of the members of the same ortholog group were searched for flanking exonic sequences. Errors in orthologous gene identification can lead to overestimate of gain and loss of introns. This is particularly a challenge for genomes that have less than ideal genome assembly (such as *Ciona*), or phylogenetically distant species (such as *Arabidopsis*). 3) Ancient paralogs resulting from gene duplication before speciation are not distinguished from recent paralogs.

We addressed some of these issues by 1) comparing genomes at various levels, starting with more closely related species and gradually adding more distantly related lineages; 2) inferring evolutionary events (intron gain, loss, and type switching) from intron clusters where all orthologous genes of compared species are present; 3) manually curating when necessary and manageable. This includes identifying homologous genes by BLAST search (tblastn), tree construction of homologous protein sequences, inspection of pairwise protein sequence alignments, and annotation of intron splice sites.

Although a U12 intron that was not present in any of the four reference genomes could be missed (and therefore not listed in any of the full set of 20 genomes), we note that the human genome, which is a U12DB reference, is also the most complete. It is unlikely that the human genome has lost more than one or two introns present in other vertebrates, because the rate of intron loss in vertebrates is low overall (see below). Furthermore, we note that almost all of the U12 introns that have been retained in fruit flies are also retained in humans and the others are missing from all chordates (see below). Thus, we feel that use of the human genome as a reference allows an accurate description of all U12 introns in vertebrates, with only a few exceptions. The one possibility we cannot rule out is high rates of U12 intron gain in some non-human vertebrate clade, but we consider this unlikely because we have observed only a single case of U12 intron gain in any species. The situation in insects is quite different. Although the *Drosophila melanogaster *genome is exceptionally complete and well-annotated, it has undergone extensive loss of U12 introns relative to other insects (see below). A thorough analysis of U12 intron evolution in insects using different methods is in progress, but not as part of the present study (Janis et al., manuscript in preparation).

### Evolution of U12-type introns in eukaryotic taxa

#### Four eutherian genomes comparison: only one intron loss

We analyzed four eutherian genomes that are of relatively good quality to gain insight into the evolution of U12-type introns through closely related species and to reduce the effect of poor data quality. According to the initial automated results, there are 442 intron clusters where the human, mouse, rat, and dog orthologous genes are all present and at least one of member intron has a "U12" status (Additional file 1, Table S1). In 407 of these 442 clusters, introns of all four species have a "U12" state. Of the remaining thirty-five clusters, seventeen have a "U12" and an "absent", seventeen have a "U12" and an "ambiguous", and a single cluster (*Syt16*) contains one "U12", one "U2", and two "ambiguous" introns. After manual inspection of the seventeen intron clusters having putative U12-type intron losses ("U12" to "absent"), only one case appears to be a true intron loss by deletion, in the gene encoding the mouse elongation factor 1 (*Elof1*). The other "absent" cases (twenty-four introns in seventeen clusters) are artefacts. Interestingly, in fourteen cases non-orthologs were compared. In the other three cases, non-cognate transcripts were compared, or genes that are present were not annotated, or were partially annotated, in the comparison genome. Thus, a case-by-case analysis reveals only two cases in which a U12 intron changes state, one loss (the second intron of the *Elof *gene in mouse) and one switch to the U2 type (the second intron of the rat *Syt16 *gene).

#### Eight vertebrate genomes comparison: eight losses and five type conversions in fish

**Figure 1:**
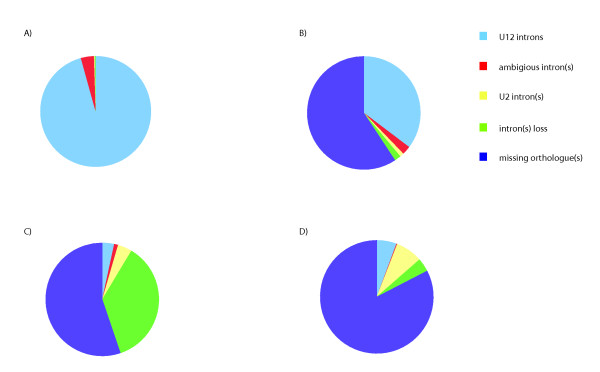
**Graphic depiction of the status of 442 human U12 intron clusters in A) Three eutheria, B) Seven vertebrates, C) *Drosphila melanogaster*, and D) *Ciona intestinalis***. The status of each intron is described as U12 in the comparison species, or in all comparison species (blue), at least one ambiguous intron in the comparison species or all comparison species (red), U2 in the comparison species or at least one of the several comparison species (yellow), missing intron (green), or missing gene - no orthologous gene detectable (violet).

We then added *Monodelphis domestica*, *Gallus gallus*, *Danio rerio*, *Fugu rubripens*, and *Tetraodon nigroviridis *to the four eutherians to compare U12-type introns within vertebrates. In this analysis, the two puffer fish (*Fugu rubripens *and *Tetraodon nigroviridis*) were represented as a puffer lineage. As more species were added to the analysis, the number of shared genes decreased. There are 180 orthologous intron clusters where orthologous genes for all vertebrates are available and there is at least one "U12" intron present in a cluster. This is considerably less than the 442 clusters in the eutherian analysis. U12-type introns are conserved in all eight lineages for 156 of the 180 intron cases. This number (156) reflects an increase from 144 after manual inspection. As in the eutherian comparison, several apparent cases of change were found to be due to ambiguous orthology or incompleteness in the genome assemblies and/or gene annotation. Furthermore, 11 of the remaining 24 cases are ambiguous, and may also represent U12 introns without change. We were able to confirm intron losses in eight clusters and U12-U2 changes in five cases. Intron state compositions of these 180 intron clusters are listed in Additional file 1, Tables S2 and S3 and depicted in Figure 1B.

Comparison of the human and *Ciona intestinalis *reveals only 77 gene clusters available for analysis. The *Ciona *intron states in these seventy-seven clusters are twenty-five "U12", one "ambiguous", seventeen "U2", and thirty-four "absent". This low conservation is likely to be exaggerated by an under-detection of tunicate U12-type introns. As mentioned earlier, the relatively poor quality of the draft genome assembly [22] and less abundant EST and/or cDNA data prevent some genes from being annotated and U12-introns from being detected. This statement is supported by the fact that 198 pairs could be compared between human and fruitfly genomes and only 154 between human and *Ciona *genomes. Nonetheless, the low number of genes compared and the high proportion of absent cases agree with the findings that the *Ciona *genome has undergone excessive loss of ancestral genes [23] and introns [24] that were present in the common ancestor of vertebrates and *Ciona*.

#### Comparison between *Homo sapiens *and *Drosophila melanogaster*

As shown in Table 1, sixteen fruitfly U12-type introns are catalogued in the U12DB. We manually examined two that have been reported earlier [25] but are missing in the U12DB and those whose hosting gene is not clustered with human genes in ensemble ortholog prediction. The protein alignment between the fruitfly sequence and the human sequence with the highest BLASTP score was used to compare intron position. Table 2 lists the adjusted status of the nineteen *D. melanogaster *U12-type introns. We were able to identify orthologous U12-type introns in the human genome for sixteen of the nineteen introns. The remaining three cases are an intron loss in early vertebrates (*SF3A1*; see Additional File 1, example 1), a case of replacement of an intron-containing gene by an intron-less gene via retroposition (*CCDC16*; see Additional file 1, example 2) and a single U2 to U12 ("twintron") conversion (*CG3294/URP*) (Additional file 1, example 3). In summary, for the 198 human genes with U12-type introns and detectable fruitfly orthologs, in the latter genome 160 introns were missing, a U2 intron in orthologous position was noted in eighteen cases, there were four ambiguous cases, and in sixteen cases U12 introns were maintained.

**Table 2:** Conservation of *Drosophila melanogaster *U12-type introns in humans.

	Annotation symbol	Termini	Gene symbol (name) in fruitflies and/or in humans	Intron number	Total number of introns	Fruitflies	Humans
1	CG6323	GT-AG	Tsp97E (Tetraspanin 97E); TSPAN13 (tetraspanin 13)	2	4	U12	U12
2	CG8408	GT-AG	TMEM41B (transmembrane protein 41B)	3	3	U12	U12
3	CG17912	GT-AG	ZNF207 (Zinc finger protein 207)	1	3	U12	U12
4	CG32705	GT-AG	ZDHHC8 (zinc finger, DHHC-type containing 8)	4	11	U12	U12
5	CG33108	GT-AG	C19orf54	2	3	U12	U12
6	CG4894	GT-AG	Ca-α1D (calcium-channel protein); CACNA1D (calcium channel)	3	31	U12	U12
7	CG7892	GT-AG	nmo (nemo); NLK (nemo-like kinase)	6	10	U12	U12
8	CG15735	GT-AG	LSM12	1	4	U12	U12
9	CG3294	GT-AG	ZRSR2 (zinc finger, RNA-binding motif and serine/arginine rich 2); U2AF (small subunit related protein)	3	4	Twintron	U2 only
10	CG16941	GC-AG	SF3A1 (splicing factor 3a, subunit 1, 120 kDa)	1	6	U12	absent
11	CG11839	AT-AC	CCDC16 (coiled-coil domain containing 16); ZNF830 (zinc finger protein 830)	1	1	U12	absent
12	CG11328	AT-AC	Nhe3; SLC9A7 (solute carrier family 9)	5	12	U12	U12
13	CG18177	AT-AC	FLJ14154 hypothetical protein	4	4	U12	U12
14	CG7736	AT-AC	Syx6 (Syntaxin 6); STX6 (syntaxin 6)	1	2	U12	U12
15	CG17228	AT-AC	pros (prospero); PROX1 (prospero homeobox 1)	2	4	Twintron	U12
16	CG15081	AT-AC	l(2)03709 (lethal (2) 03709); PHB2 (prohibitin 2)	3	5	U12	U12
17	CG3427	AT-AC	Epac; RAPGEF3 (Rap guanine nucleotide exchange factor 3)	11	16	U12	U12
18	CG11984	AT-AC	KCMF1 (potassium channel modulatory factor 1)	3	6	U12	U12
19	CG15589	AT-AC	Ca-alphaT-RC (Voltage-dependent T-type calcium channel)	1	23	U12	U12

Depending on the terminal dinucleotides, or termini, U12-type introns are divided into two major subtypes. Of the nineteen fruit fly U12-type introns, ten are GT-AG subtype, including one GC-AG and eight AT-AC. Sixteen have U12-type ortholog in humans. These sixteen human orthologous U12-type introns are of the same subtype as their fruitfly ortholog. The absence of the U12-type intron in the human SF3A1 gene is likely an intron deletion after insects diverged. The *CCDC16 *gene is intronless in mammals and fish but not in chickens and frogs. Thus, the absence of the U12-type intron in the human *CCDC16 *gene is presumably due to a retroposition of the gene that occurred in early mammals. The third intron of the fruit fly *CG3294 *(URP) gene has two pairs of splice sites that form two alternative spliced introns, one of the U12-type and one of the U2-type. The pair of U12-type splice sites is absent in non-diptera species.

Fifteen orthologous mammalian introns have the same terminal dinucleotides as their fruitfly orthologs, the only exception is the one with GC-AG in *Drosophila *(which is GT-AG in mammals). Fish orthologs have an additional subtype switch from AT-AC to GT-AG in the *Syntaxin-6 *gene. We also examined orthologs of *D. melanogaster *U12-type introns in twelve drosophila and one mosquito genome whenever data was available. Interestingly, we found a case of two independent switches from a U12-type to a U2 intron in the *ZDHHC8 *gene. This intron is of the U12-type in vertebrates and most dipterian species, but of the U2-type in *D. ananassae *and *Anopheles gambiae*.

#### Comparison between *Homo sapiens *and *Arabidopsis thaliana*

Even though more than 200 U12-type introns were identified in *A. thaliana*, only two of them are reported by U12DB to have orthologous U12 introns in humans. These two introns are in genes encoding exosome component 5 (*EXOSC5*) and BRCA1/BRCA2-containing complex, subunit 3 (*BRCC3*). Most Arabidopsis U12 introns are in genes that are reported to lack human orthologs. Twenty-five *A. thaliana *U12-type introns are in genes with human orthologs but no orthologous intron, and another twenty-eight have orthologous genes (but not introns) in non-human animals. If these numbers are correct, the number of *A. thaliana *U12-containing genes that have human orthologous genes is underrepresented compared to the number of *A. thaliana *genes that have human orthologous genes (p-value 7e-17 by the hypergeometric distribution test, based on clustering data from InParanoid, a database of Eukaryotic Orthologs [26]). However, a recent analysis by Basu *et al. *[21] reported twenty U12-type introns shared between humans and *Arabidopsis*. This is therefore likely to be a more extreme case of what was observed for *Drosophila*, i.e. the U12DB fails to track orthologs over greater evolutionary distances.

#### Summary of comparisons within various clades

The above analyses on various subsets of genomes were based on relatively conservative datasets; intron clusters were not considered for those genes where orthologous gene information is missing for any of the species compared. In summary, introns are classified as U12-type in all four eutheria for 423 (95.7%) of the 442 clusters (unique intron positions) where four eutherian orthologous genes are present. Seventeen clusters contain ambiguous introns, indicating potential conversion, but as many as 440 of the 442 clusters may be U12-type in all genomes considered. There is only one unambiguous case of U12 intron deletion (*Elof1 *in the mouse genome) and one potential U12-U2 transition (*synaptotagmin XIV *in rats). Among the eight vertebrates, in 156 (86.7%) of the 180 clusters, U12-type introns are conserved in all eight lineages. There are intron losses in eight clusters and U12-U2 conversion in five clusters. Eleven clusters contain ambiguous introns. No gain of a U12-intron was observed. These results indicate that U12-type introns have been tremendously stable in vertebrates during the last 550 million years after the divergence of *Ciona*. There were only a few losses or U12-U2 conversions. Conservation between *Ciona *and vertebrates is certainly underestimated mainly due to the relatively poor quality of the *Ciona *genome draft, which leads to under detection of U12-type introns and difficulties in ortholog prediction. The conservation of U12-type introns between humans and fruitflies is very low (7.5% of 200 introns) since there are only nineteen U12-type introns in *D. melanogaster*. Fifteen of these have orthologs in humans; one is specific to diptera, and two are lost in humans (mammals).

#### Massive loss of U12-type introns in invertebrates

Depending on availability of data and phylogenetic distance between examined genomes, the number of analyzed intron clusters varied from 77 to 442, which account for at most two thirds of the near 700 metazoan U12-intron clusters (unique intron positions) in the U12DB. Since only fourty-seven intron clusters contain non-chordate U12-type introns, obviously, the modern-day U12-type introns identified in extant animals are mostly limited to vertebrates. To learn how these orthologous vertebrate U12-type introns (those that are present in the human and at least one non-primate genomes) relate to other lineages, we investigated their conservation pattern from a broader and more inclusive perspective.

We were able to find invertebrate orthologous genes for 267 vertebrate genes that contain a U12 intron. In 194 cases the orthologous intron was missing from the invertebrate gene. Although the data does not provide direct evidence whether these are intron gains in vertebrates or losses in non-chordates, the following observations suggest that U12-type intron loss is more frequent than U12-type intron gain. First, our four eutherian and eight vertebrate analyses did not uncover a single case of U12-type intron insertion. Second, as mentioned above, the minor-type splicing system independently went extinct in some organisms via intron deletion and/or conversion to U2-type, indicating that the loss rate of U12-type introns is greater than gain rate and that the entire system has been lost repeatedly [14]. Third, by studying thirty gene loci from the marine annelid (*Platynereis dumerilii*), *C. elegans*, insects, ciona, and humans, Raible et al. [24] reported that genes in Urbilateria, the common ancestor of worms, insects, tunicates, and humans, are "vertebrate-type" i.e. intron-rich and that the disparity in intron abundance between invertebrates and vertebrates is mainly due to an intron loss in invertebrates. Fourth, with apparent lower intron abundance and density than other higher eukaryotes, the *D. melanogaster *genome appears to have undergone extensive intron loss [24,27-29]. A study using a maximum likelihood approach infers that the ratio of intron loss rate over gain rate (for all introns) is 1.25/0.01 = ~125 in *D. melanogaster*, 1.04/0.02 = ~50 in *C. elegans*, 0.77/0.04 = ~20 in Ciona, and 0.35/0.21 = ~2 in humans [29]. These ratios are likely to be greater for U12-type introns, assuming the same intron gain mechanism for both types of introns. The loss rate of U2-type introns in various species agrees with the prediction from a population genetics perspective. Lynch and Richardson [15] argued that harbouring introns imposes deleterious effects because errors in splicing can lead to a failure in producing functional proteins. The larger the effective population size of a species, the more effectively selection will remove introns. This explains the intron-rich vertebrates and intron-poor invertebrates and microbes. The loss rate may be even higher for U12-type introns because hosting U12-type introns can be more deleterious than hosting U2-type introns. The primary argument for this is that the requirement for an extended recognition motif makes U12-type introns more susceptible to mutations. Other factors that have been proposed to have a negative impact include the slower processing of U12-type introns [30] and higher splicing error rates [17,31]. Therefore, selection against U12-type introns may be greater than selection against U2-type introns. The disparity in the number of U12- and U2-type introns may be greater in species with large populations than ones with small populations. Thus, U12-type introns have been steadily lost in nematodes and insects, species with historically large population sizes, but maintained among vertebrates, which have historically smaller population sizes.

#### Nearly half of vertebrate genes with U12-type introns are vertebrate-specific

Of the 549 clusters with a U12-type intron present in humans and at least one non-primate vertebrate, invertebrate orthologous genes are absent in 254 of them. Thus, not only are the majority of U12-type introns found in metazoans present in vertebrates but also the genes that harbour them are often vertebrate-specific. While some of the 254 cases of vertebrate-specific genes might be due to extensive gene losses in invertebrates [23,32], others may have resulted from the large scale segmental duplication or whole genome duplication (WGD) event(s) that have been proposed to take place in early vertebrate evolution [33,34]. The two gene families that encode the alpha subunit of voltage-gated sodium and calcium channels, SCN and CACN1, respectively, illustrate this.

Mammals have eleven sodium and ten calcium channel alpha subunit genes while fruitflies have two and three, respectively [35-37]. Most mammalian *SCN *and *CACN1 *genes have two U12-type introns [12,38,39], however fruitflies preserved only one U12-type intron in one of the calcium channel gene. Human and mouse *SCN *genes are located in four chromosomal regions that, interestingly, also harbour *HOX *genes. This suggests that the large-scale segmental duplications and subsequent tandem gene duplications led to the expansion of *SCN *genes in mammals [37].

#### Metazoan U12-type introns are more often lost than converted to U2-type introns

A unique aspect of the evolution of U12-type introns is the conversion from a U12- to a U2-type which was first proposed by Burge *et al. *[13]. Both intron deletion and U12 to U2 conversion reduce the abundance of U12-type introns in a genome. Thus, an interesting question is how these two mechanisms contribute to the loss, or even the extinction, of U12-type introns in invertebrates. Assuming that intron type conversion is unidirectional and U12 intron gain is negligible [13], invertebrate U2-type introns orthologous to vertebrate U12-type introns represent U12 to U2 conversions. When we look at orthologous positions of human U12 introns in different species some trends are apparent. The ratios of absence vs. U2-type introns in these positions are 2:1, 9:1, and 5:1 for tunicates, fruitflies, and nematodes, respectively (see Table 1). This suggests that deletion contributes more than U12-U2 conversion to the evolution of U12-type introns in metazoa, although the numbers of cases observed do not directly reflect the rates of the two processes, e.g. some U12-turned-U2-type introns might have been lost and are not counted. This might be because a U12 to U2 conversion requires multiple fortuitous mutations or circumstances. It has been shown that nucleotide substitutions at positions +4 to +7 of a U12-type 5' splice site, particularly a C to G change at +5, can activate cryptic splice sites in the vicinity, leading to splicing through U2-type pathway, or leave the intron not removed [12,40]. Such mutations are more likely to result in non-functional proteins, and in turn, the mutant alleles are quickly removed from the population. Moreover, if the initial mutation does not disrupt the reading frame, the intron is likely to become an ambiguous intron that is processed inefficiently by either spliceosome. Subsequent mutations would typically be required to improve the splice site of the U12-turned U2-type introns. Hence, the transition from a U12-type to a U2-type requires multiple changes and intermediates are likely to be mutant alleles whose frequency will drift in the population. On the other hand, intron deletion is less likely to disrupt the reading frame of the mature mRNAs, and thus has a less negative effect than nucleotide mutations in splice sites. In turn, alleles with a deleted intron are more likely to be retained and fixed more quickly.

#### Gene retrotransposition and consequent loss of introns

We have observed several cases of U12 intron loss that can be attributed to retrotransposition. In vertebrates, where U12-type introns are extremely conserved, gene retroposition appears to be responsible for three out of the eight losses of U12-type introns (the affected genes are intronless). Interestingly, *CCDC16*, the vertebrate ortholog of the *D. melanogaster *U12-containing gene *CG11839*, has undergone three independent retropositions followed by a loss of intron-containing alleles, one in early mammals and two in distinct fish lineages. Only thirteen of the 160 *D. melanogaster *orthologs of the vertebrate U12-containing genes are intronless. However, retrotransposition followed by intron gain is more likely at such a long phylogenetic distance, so we cannot rule out a larger role of retrotransposition in U12 intron removal.

#### U12-U2 conversion occurs more frequently in GT-AG than AT-AC subtype

Of the nearly one thousand U12-type intron clusters in the U12DB, eighty-seven clusters have both U12- and U2-type introns in orthologous position. These eighty-seven non-redundant U12-type introns can be presumed to have undergone type-conversion at least once during the course of evolution. Eight of them also have both of the U12 subtypes. Of the remaining seventy-nine clusters, seventy-four are of the GT-AG subtype and five are of the AT-AC. The ratio of GT-AG to AT-AC subtypes in various genomes catalogued in the U12DB ranges between 3:1 and 1:1. Comparing this ratio to the total number of clusters of each subtype, 565:204 (GT-AG:AT-AC), indicates that the underrepresentation of the AT-AC subtype among clusters with conversions is statistically significant (P-value 0.000015 from a Fisher Exact test). Moreover, all eighty-seven U2-type introns involved in conversion have GT-AG termini. This suggests that GT-AG U12-type introns are much more likely to be converted to a U2-type. There are thirty-six clusters with U12-introns with both subtypes indicating subtype switching. Thus, it seems likely that AT-AC intron conversion to the U2-type is preceded by a subtype switching from AT-AC to GT-AG, as previously proposed by Burge *et al. *[13].

#### A rare pathway from AT-AC U12 to AT-AC U2-type

Although most type conversions from an AT-AC U12-type are likely to involve prior subtype switching, we think that the existing AT-AC U2-type introns are the outcome of a much more rare U12 to U2 conversion pathway. These AT-AC U2-type introns in the sodium channel genes all have a G at position +5, a feature that has been shown to be important for recognition of the 5' splice site by the U2-spliceosome [12]. A possible scenario is that a C to G mutation at position +5 turns an AT-AC U12-type intron into a suboptimal U2-type. Subsequent mutations, particularly to an A at position +4 or a T at position +6, would improve the recognition of this site by the U2 spliceosome. The resulting AT-AC U2-type intron may then be "trapped" because, unlike in the U12-type splicing, the U2-type splicing allows less flexibility in the non-Watson-Crick interaction between the first and last nucleotides. Subtype switching would require simultaneous mutations at the intron termini, while conversion to the AT-AC U12 type would require multiple simultaneous mutations within the 5' splice site itself. These difficulties may explain the persistence of suboptimal AT-AC U2-type introns.

#### Non-canonical U12-type introns

Given that the 5' end dinucleotides of U12-type introns seem to be under less constraint than are U2-type introns, we investigated non-canonical 5' dinucleotides present in U12-type introns. In the U12DB, nineteen U12-type introns do not have canonical donor sites (GT, GC, or AT). Manual examination revealed that six are annotation errors (four have AT-AC termini and two have GT-AG). Six of the remaining thirteen are unambiguous U12 non-canonical 5' dinucleotides. There are two CT-ACs in the mouse and rat genes encoding trafficking protein particle complex 9 (*Trappc9*, *Nibp*), three GG-AG introns in the macaque and cow *SLC12A4 *genes and the rat *SLC25A30 *gene, and one GA-AG in the zebrafish *actr10 *gene. In all of these cases, orthologous introns in other vertebrates have either AT-AC or GT-AG consensus dinucleotide termini. Naturally occurring U12-type introns with these three sets of termini (CT-AC, GG-AG, GA-AG) have never been reported before. In a mutational study of terminal dinucleotide in U12-type introns, all sixteen possible combinations of first and last nucleotides were tested in the context of NU-AN [41]. Indeed the combination CU-AC yields correct splicing products, albeit with a lower efficiency than the wide type. Claiming that the U at the second and the A at the one next to the last position of an intron (N**U**-**A**N; denoted as +2 at the 5' end and the -2 at the 3' end, respectively) are highly conserved according to database search and mutant analyses; the authors also tested the functionality of twelve combinations (excluding G2) of nucleotides at these two positions in the context of AN-NC. The results suggested that the nucleotides at +2 and -2 do not interact in the same way as those at +1 and -1. Therefore, no experimental splicing data appear to challenge the functionality of GG-AG and GA-AG termini.

The discovery of these non-canonical U12-type introns has two important implications. Firstly, this finding shows that the variation in the 5' termini is greater than previously thought and confirms the results of that mutational study [41]. Secondly, it underscores the difficulty faced if noncanonical introns are to be recognized in genome-scale analyses.

## Conclusions

We investigated conservation and changes of the intron state among orthologous U12-type introns identified in eighteen metazoan genomes. From our study it is clear that intron loss contributes much more than conversion of U12- to U2-type introns to the evolution of U12-type introns. Conversion occurred more frequently in the GT-AG subtype of U12 introns than in the "classic" AT-AC subtype. Intron subtype switch has been widely regarded as a unidirectional process [23,21]. However, this picture has to be revised in the light of our discovery of the new dipterian U12-type intron in the *dURP *gene. Interestingly, this intron appears to be a twintron with ancestral U2-type splicing signals still active. With the presence of these splice sites, the U12-type cis-elements arose from activation of cryptic splice site that can be dated to early diptera. We also discovered six non-canonical U12-type introns with three sets of terminal dinucleotide that have never been reported before (CT-TC, GG-AG, and GA-AG). This indicates that U12-type 5' termini are more degenerate than previously thought. At most, one case of U12-type intron gain was found.

The presence of orthologous U12-type introns among vertebrates has been highly conserved in the past 550 million years. The conservation is especially marked among eutheria. Of the 442 unique intron positions, only one unambiguous loss in mice and one U12 to U2 conversion in rats were observed. The non-chordates investigated have considerably fewer U12-type introns than vertebrates. Interestingly, the lineage with the fewest U12 introns, diptera, is the only one that acquired a new U12-type intron. Although there is no orthologous intron from outgroups in most of the nearly 200 cases of intron loss several facts support intron loss view. Nearly half of vertebrate U12-containing genes are absent in non-chordates. This is likely a combination of gene losses in the non-chordates and neofunctionalization or subfunctionalization of genes arising from large-scale segmental duplication or whole genome duplication that occurred in early vertebrates, as is exemplified by the multiple-U12-containing genes that encode the ion channel alpha subunit. Thus, while our summary clearly shows that all changes occur only slowly, it appears that the loss of U12 introns greatly outstrips the gain of U12 introns. We find only a single clear-cut case of U12 intron gain. We note the recent exciting discovery of high rates of intron gain in the crustacean *Daphnia *[42]. It will be interesting to learn whether U12 introns are also being gained in that species. Finally, recent availability of more than twenty insect genomes creates an ideal platform for studying the evolutionary dynamics of U12-type introns. Presently, we are taking advantage of this system to investigate in detail what steps are necessary to switch from one intron type to another and what functional consequences it may have.

## Methods

### Data

We downloaded the MySQL version of the entire U12DB version 1.0 published by Alioto [43]ftp://genome.imim.es/pub/software/u12/u12db_v1_0.sql.gz. U12DB, a database with a web interface, provides information for U12-type intron identified in twenty sequenced eukaryotic genomes and clusters of U12-type introns and orthologous introns. In U12DB, orthology between two introns was determined by sequence similarity of their flanking exonic sequences. Each orthologous U12-type intron cluster represents a unique intron position with respect to the protein sequence that is derived from the hosting genes. At least one of the member introns is of the U12-type. Therefore, a set of orthologous genes can share more than one U12-type intron cluster.

Statuses of the introns in a cluster were encoded with "1" - U12-type, "2"-ambiguous, which is denoted as "U12/U2" in the U12DB, "3" - U2-type, and "4" - intron absent. Intron states of all intron clusters were converted into a matrix of intron status versus species. Each orthologous intron cluster is represented in the matrix as one line of intron states for all species analyzed. If gene orthology information is lacking for a certain species, the state of this species is coded by a "5" - missing data. On the other hand, if multiple genes from one species are present in one cluster, intron states of the same species were ranked in the order of U12-type, ambiguous, U2-type introns, and intron absent. The top ranking was retained to represent the species. Patterns of intron states were used to infer conservation of the U12-type intron among taxa at varying phylogenetic distances. We conducted manual inspections extensively to correct potentially false cases of intron absence and missing orthologs in the U12DB. We relied on a variety of sources for validation of automated results when necessary. These sources included Entrez Gene [44], evidence viewer, and Blink from NCBI, Ensembl [45], the UCSC Genome Browser [46], and the UniProt Knowledgebase [47].

## Authors' contributions

CL carried out the acquisition, analysis, and interpretation of data, and drafted the manuscript. SM participated in analysis and interpretation of data, and writing. AJ conceived of the study and participated in interpretation of data. WM coordinated and designed the study, participated in analysis and interpretation of data, and writing. All authors read and approved the final manuscript.

## Supplementary Material

Additional file 1 **Here we present several examples of the evolutionary fates of specific U12-type introns.** These examples illustrate the modes of evolutionary changes for U12 introns. Click here for file
