# Supplementary material for: Evolutionary dynamics of U12-type spliceosomal introns
Source: BMC Evol Biol. 2010 Feb 17;10:47. doi: 10.1186/1471-2148-10-47 (PMC2831892; doi:10.1186/1471-2148-10-47)
Supplement: Additional file 1 — Here we present several examples of the evolutionary fates of specific U12-type introns. These examples illustrate the modes of evolutionary changes for U12 introns. [file 1471-2148-10-47-S1.PDF]

# Supplemental Materials

## Illustrative examples of the evolution of U12-type intron

Here we present several examples of the evolutionary fates of specific U12-type introns. These examples illustrate the modes of evolutionary change for U12 introns.

### Example 1: An insect-specific U12-type intron in the *SF3A1* coding gene

There are six introns in *D. melanogaster*'s *SF3A1* gene, which encodes for subunit 1 of splicing factor 3a. The first one is a U12-type intron with non-canonical GC-AG termini. This intron is present in all twelve *Drosophila* genomes recently sequenced [1]. The orthologous introns in the honeybee (*Apis mellifera*) and beetle (*Tribolium castaneum*) genomes are also of the U12 type, but have canonical GT-AG termini. However, mosquito and *Caenorhabditis elegans* genomes have a U2-type intron that is missing from the orthologous gene in plants and all vertebrate genomes that we examined. A reconstructed evolutionary history inferred from states of orthologous introns and based on the coelomata hypothesis is shown in Figure S1, on the left. Here, an ancestral metazoan U12-type intron was converted to a U2-type in nematodes (non-coelomata), and was lost in deuterostomes (represented by four vertebrates here) after the speciation of insects (arthropods). The U12-type intron in early insects was later converted to a U2-type in one dipteran lineage (mosquitoes). Independently, GT to GC substitution occurred in the 5' dinucleotide in another dipteran lineage. Another scenario, based on the alternative phylogeny where nematodes and arthropods form the ecdysozoa clade, would be that the U12-type intron arose after divergence of the dueterostomes. The succeeding events were the same as those inferred according to the coelomata hypothesis except that there was no loss event in deuterostomes. The latter scenario is more favourable from a parsimony point of view because it requires fewer evolutionary steps to explain the current status of this particular intron in different metazoan lineages, but one of those events (the gain of a U12 intron) appears to be rare.

One of the five major components of the U2-type spliceosome is the U2snRNP, which is built up by an snRNA and several polypeptides, including subcomplexes SF3B, which is also present in the U11/U12 snRNP [Will et al. 2004] and SF3A, which is not [2]. SF3A1, whose gene contains a U12 intron, is therefore a component of a U2-specific splicing system. Thus, there is an interesting relationship between two splicing systems. That is, formation of a U2-type spliceosome requires protein products involving the U12-dependent splicing pathway - removal of the U12-type intron from SF3A1 pre-mRNAs.

**Example 2: U12-type intron loss by retroposition**

The *D. melanogaster* gene *CCDC16* (coiled-coil domain containing sixteen) contains a single intron that is of the U12 type with AT-AC termini. However, the gene entries in the flybase (gene ID CG11839) and the RefSeq (NM_170198; gi 28571873) are incorrectly annotated with a U2-type 5' splice site twenty-six nucleotides upstream of the real intron/exon boundary. The U2 intron is not supported by any data but the U12 is supported by a single cDNA sequence. Moreover, the current RefSeq record replaced a correctly annotated version of the gene (NCBI gi 24649914). Plant orthologs *(A. thaliana* and *Oryza sativa)* contain an orthologous U2-type intron. Interestingly, in many vertebrate orthologs are intronless, e.g. all placental mammals and fish. Yet, other vertebrates, including frogs, lizards, chickens, zebrafish, platypuses, and lampreys, have CCDC16 genes with multiple introns. In these genomes, a U12 intron orthologous to the one in Drosophila is apparently present among other U2 type introns. This supports the hypothesis that the ancestral vertebrate possessed a multi-intronic *CCDC* gene and two independent retroposition events depleted the gene of introns in early mammals and early fish lineages.  We were not able to find an intron-containing copy of the *CCDC16* gene in the genomes that have a likely retrotransposed version of the gene. In short, in all the genomes studied there is always a single copy of the gene with no paralog detectable.

**Example 3: U12-U2 conversion and neofunctionalization in *XDH* and *AOX1* genes**

The genes encoding xanthine dehydrogenases (*XDH*) and aldehyde oxidase (*AOX1*) are apparent homologs. In the human genome they share a similar intron/exon structure and the encoded amino acid sequences are fifty percent identical. These proteins have similar enzymatic activities; while *XDH* is involved in purine catabolism through urate, *AOX1* uses aldehyde substrates. Their activities have been located to peroxisomes and to cytoplasm, respectively. In all studied mammalian genomes, the second and the third of a total thirty-four introns in the human *XDH* gene are of the U12-type. However, in the *AOX1* gene the second intron remains to be of the U12-type, but the third one has been converted into the U2-type. The same pattern exists in all studied mammalian genomes.

While the overall gene structure varies from nematodes (fifteen introns) to vertebrates (thirty-five introns), introns at the same postion as the two human *XDH* U12-type introns remain present across all *XDH* and *AOX* genes studied, with an exception of fruit flies, where only three introns are preserved. These introns are both U2-type in *C. elegans* and *Ciona,* but reveal an interesting pattern among vertebrate *XDH* and *AOX1* genes (see Figure S2). The second and the third intron of *XDH* and *AOX1* genes are U12-then-U12 and U12-then-U2, respectively, in all mammals, and ambiguous-then-U12 and U12-then-U12, respectively, in chickens. Assuming that in the ancestral gene both introns were U12-type, one can conclude that there was a U12- to U2-type switch of *AOX1*'s third intron in the common ancestor of mammals. The switch seems to have been triggered by deletion of a thymine at position +4 of the 5' splice site and was followed by a substitution and/or deletion at the U12 BPS. Both intron 2 and 3 of the *AOX1* gene are U12-type in two pufferfish and chickens but are of the U2-type in zebrafish. This suggests that the type switch in zebrafish is independent from the one mentioned above in early mammals. The second intron of *XDH* is a U2-type in two pufferfish but a U12-type in zebrafish and ambiguous in frogs and chickens. Intron type switch in *XDH* and

*AOX1* genes does not seem to have any impact on the protein products and no evidence directly links the intron type change to neofunctionalization of the *AOX1* gene.

**Example 4: Two U12-type introns are present in VPS16 in most genomes but both are missing in zebrafish**

As mentioned above, three of the eight U12-type intron losses in the fish lineage are likely due to gene retroposition (Table S3); U12 introns were lost together with all the other introns in the gene. For the remaining five losses, three occurred in three pufferfish genes while two occur in a single zebrafish gene, *VPS16*, which encodes vacuolar protein sorting 16. The gene is believed to encode proteins that may play a role in lysosomal delivery of vesicle-mediated proteins and contains twenty-three introns in the human genome. Introns 9 and 13 are both U12-type. A rare isoforms couples the donor site of intron 9 with the acceptor of intron 13 such that four exons and five introns are removed as a single U12 intron (Figure S3). This leads to a deletion of 144 aa in the protein, but it is not clear whether this isoform is functional or not. Several independent studies, including cloning and characterizing four human VPS genes [3], found ubiquitous expression of the longer isoform. Another study characterizing full-length human cDNAs [4], however, reported expression of the shorter isoform only in kidneys. This seems to suggest that isoform 1 is expressed ubiquitously but expression of the isoform 2 is tissue specific and the regulation of alternative splicing is likely to be involved with the minor spliceosome.

Interestingly, orthologs of these two U12-type introns are both missing in the *Danio rerio* VPS16 gene while the remaining twenty-one introns are all in place. Mechanisms for causing intron deletion that have been proposed/known to date [5] predict that introns are lost either as single introns (by genomic deletion) or multiple adjacent introns (mediated by reverse-transcribed-mRNA). Deleting two nonadjacent introns would require more than one deletion event. It is striking in the case of the *Danio rerio* VPS16 gene that the targets of two events are both U12-type introns (two out

of twenty three). The straightforward explanation is that the two U12-type introns were lost in two independent events. Alternatively, a partially spliced precursor from which only the U12 introns have been removed might have served as a donor for retrotransposition.

**Example 5: A U12-type intron in *Drosophila* CG3294 (URP)**

Two splicing variants (CG3294-RA and CG3294-RB) are annotated for the *D. melanogaster* CG3294 locus. These annotations are based on a combination of EST data and sequence similarity to the human U2AF35-related protein (*URP*) sequence, which contains a divergent CCCH zinc finger RNA-binding motif and an RS (aRginine/Serine) domain. The HGNC gene symbol for this human URP gene is ZRSR2. Here, we denote the *Drosophila* and human URP as dURP and hURP, respectively. CG3294-RB, which is supported by EST evidence, has a U12 intron. CG3294A is generated from an overlapping U2-type intron whose 5' splice site is 29 nucleotides upstream and whose 3' splice site is 30 nucleotides upstream of the U12 sites. The extended 29-nucleotide exonic sequence specific to CG3294-RB introduces a pre-mature stop codon, which truncates the protein (314 vs. 456 amino acids). As a result of this pre-mature stop codon, CG3294-RB mRNA is predicted to be a target for degradation by the mRNA surveillance system, Nonsense-mediated mRNA Decay (NMD). Indeed, transcripts from this gene are stabilized between 1.4 and 1.8-fold by mutations in factors required for NMD. A similar alternative splicing pattern has been observed in many SR protein genes [6-7].

To learn the evolution of the *dURP* U12-type intron, we looked for the orthologous gene and the transcript (only the regions flanking the U12-type were considered) that is cognate to the dURP U12-isoform in other organisms. While the *URP* genes in twelve drosophila and two mosquito genomes contain the orthologous U12-type intron and presumably they can express both the U2- and the U12-isoform, those in all other animal genomes seem to express only the U2-isoform (see Figure

S4). Thus, it is clear that the U2-isoform represents the ancestral state and the U12-isoform (with the U12-type intron) was newly acquired in the common ancestor of diptera.

Furthermore, there must be a full-length protein (product of the U2-isoform), and a "truncated" version (product of the U12-isoform). A novel *dURP* isoform that allows it to be post-transcriptionally regulated by nonsense-mediated decay is a significant evolutionary event in several aspects. First, no other *de novo* U12-type splicing sites have been documented so far. Second, the dipteran U12 intron within the *URP* gene demonstrates that U12-type introns can arise via alternative splicing, as opposed to intron insertion. Third, a dipteran *URP* U12-type intron reveals that in genomes where U12-type introns are so scarce (fewer than twenty in drosophila), new U12-type introns can still arise. Four, the drosophila *URP* gene demonstrates a "reverse" pattern of alternative splicing to that of the well studied drosophila *prospero* gene [8] where a newly arisen and alternatively spliced U2-type intron is embedded in an ancestral U12-type intron (a so-called twintron arrangement). This has been proposed to be one of the pathways whereby U12-type introns were lost in drosophila [9].

Interestingly, similar to the *dURP* U2-isoform, the mosquito (*Anopheles gambiae*) U2-isoform is also predicted based on sequence similarity to *hURP* without EST/mRNA support, while the U12-isoform is not annotated even though it is supported by ESTs. Therefore, numbers of EST/mRNA sequences detected for the *Drosophila* and mosquito *URP* genes seem to suggest that the *URP* U12-isoform is more abundant than the U2-isoform in both diptera. The fact that AS-NMD was newly acquired by the *dURP* but not the *hURP* gene is consistent with the finding that the majority of NMD targets in *Drosophila* are not orthologous to those in humans [7]. Thus, even though NMD surveillance has been found in many eukaryotes, regulating ten to twenty percent of the transcript in addition to preventing the translation of mRNAs that contain non-sense mutation [10], genes become the target of NMD as a means of regulating expression independently in different species.

## References


1. Clark AG, Eisen MB, Smith DR, Bergman CM, Oliver B, Markow TA, Kaufman TC, Kellis M, Gelbart W, Iyer VN, et al: **Evolution of genes and genomes on the Drosophila phylogeny.** *Nature* 2007, **450:**203-218.

2. Will CL, Schneider C, Hossbach M, Urlaub H, Rauhut R, Elbashir S, Tuschl T, Luhrmann R: **The human 18S U11/U12 snRNP contains a set of novel proteins not found in the U2-dependent spliceosome.** *RNA* 2004, **10:**929-941.

3. Huizing M, Didier A, Walenta J, Anikster Y, Gahl WA, Kramer H: **Molecular cloning and characterization of human VPS18, VPS 11, VPS16, and VPS33.** *Gene* 2001, **264:**241-247.

4. Ota T, Suzuki Y, Nishikawa T, Otsuki T, Sugiyama T, Irie R, Wakamatsu A, Hayashi K, Sato H, Nagai K, et al: **Complete sequencing and characterization of 21,243 full-length human cDNAs.** *Nat Genet* 2004, **36:**40-45.

5. Roy S, Gilbert W: **The evolution of spliceosomal introns: patterns, puzzles and progress.** *Nat Rev Genet* 2006, **7:**211-221.

6. Lareau LF, Inada M, Green RE, Wengrod JC, Brenner SE: **Unproductive splicing of SR genes associated with highly conserved and ultraconserved DNA elements.** *Nature* 2007, **446:**926-929.

7. Rehwinkel JAN, Letunic I, Raes J, Bork P, Izaurralde E: **Nonsense-mediated mRNA decay factors act in concert to regulate common mRNA targets.** *Rna* 2005, **11:**1530-1544.

8. Scamborova P, Wong A, Steitz JA: **An Intronic Enhancer Regulates Splicing of the Twintron of Drosophila melanogaster prospero Pre-mRNA by Two Different Spliceosomes.** *Mol Cell Biol* 2004, **24:**1855-1869.

9. Mount SM, Gotea V, Lin C-F, Hernandez K, Makalowski W: **Spliceosomal small nuclear RNA genes in 11 insect genomes.** *Rna* 2007, **13:**5-14.

10. Conti E, Izaurralde E: **Nonsense-mediated mRNA decay: molecular insights and mechanistic variations across species.** *Current Opinion in Cell Biology* 2005, **17:**316-325.

## Supplemental figure legends

Figure S1. The GC-AG U12-type intron in the splicing factor 3a subunit 1 (SF3A1) coding gene from *D. melanogaster* and its orthologous intron. The reconstructed evolutionary history based on the ceolomata hypothesis is presented in the left panel and the one based on the ecdysozoa hypothesis in the right panel. Species/taxon names are color-coded for the status of the intron of interest: red - U12-type, blue - U2-type, and black – intron absence. Branch lengths are not drawn to scale. Note that in the ecdysozoa scenario, the loss event in deuterostomes is unnecessary. X denotes intron loss and % denotes U12 to U2 intron type conversion.

Figure S2. The two U12-type introns in *AOX1* and *XDH* genes. Abbreviated intron sequences from *AOX* and *XDH* genes from selected species are shown. Assuming that the ancestral state of both introns is U12, the third intron of AOX underwent a U12-to-U2 type switch in the ancestor of mammals. The switch of both AOX introns to U2 in zebrafish (*D. rerio*) must have occurred independently. In the case of XDH, the U12-to-U2 switches of intron 2 in pufferfish, and both introns in Ciona and C. elegans are likewise independent.

Figure S3. Two U12-type introns in the human *VPS16* gene are missing in the zebrafish ortholog. (a) The human *VPS16* gene has two splicing variants (isoform 1 - NM_022575 and isoform2 - NM_080413). In isoform 1 two U12-type introns (intron 9 and 13 denoted by red line) and three U2-type introns (intron 10, 11, and 12) are being removed. In isoform 2 the donor site of intron 9 couples the acceptor site of intron 13, and thus, the five introns and four exons form a long intron. This results in the skipping of four exons (exon 10, 11, 12, and 13) that encode 144 amino acids. (b) Both of the U12-type introns are missing in the zebrafish VPS16 gene.

Figure S4. The GT-AG U12-type intron in the U2AF35-Related Protein (URP) coding gene. A) Phylogenetic distribution of the *URP* gene and the protein alignment of the flanking regions. The *URP* gene has one splice variant (isoform A) in most lineages. In fruitflies and mosquitoes it produces a distinct isoform (isoform B) resulted from removing a U12-type intron. Excision of the U12-type intron introduces a premature stop codon and leads to a unique 3’ untranslated region. B) Nucleotide sequences and derived protein sequences surrounding the two junctions. Light blue boxes represent coding exons and brown boxes represent UTRs. Blue lines represent a U2-type intron and red lines represent a U12-type intron.

## Supplementary tables.

Table S1. Intron state composition of eutherian orthologous U12-type intron clusters. Each orthologous intron cluster represents a unique intron position. ambi: ambiguous (denoted as U12/U2 in the U12DB)

| ***H. sapiens*** | ***M. musculus*** | ***R. norvegicus*** | ***C. familiaris*** | **Number of clusters** |
|---|---|---|---|---|
| U12 | Lost[1] | U12 | U12 | 1 |
| U12 | ambi | U2[2] | ambi | 1 |
| U12 | U12 | U12 | U12 | 423 |
| U12 | U12 | U12 | ambi | 4 |
| U12 | U12 | ambi | U12 | 2 |
| U12 | ambi | U12 | U12 | 3 |
| U12 | ambi | ambi | U12 | 1 |
| U12 | ambi | ambi | ambi | 1 |
| ambi | U12 | U12 | U12 | 2 |
| ambi | U12 | U12 | ambi | 1 |
| ambi | U12 | ambi | ambi | 1 |
| ambi | ambi | U12 | ambi | 1 |
| ambi | ambi | ambi | U12 | 1 |

[1] The second intron of the human *ELOF1* (Elongation Factor 1) gene is lost in the mouse genome.

[2] Second intron of rat *Syt16* (synaptotagmin XIV-like) gene.

Table S2. Intron state composition of vertebrate orthologous U12-type intron clusters. Eutheria include *H. sapiens*, *M. musculus*, *R. norvegicus*, and *C. familiaris*; puffers include *F. rubripes* and *T. nigroviridis*.

| eutheria | M.domestica | G.gallus | D.rerio | puffers | clusters |
|---|---|---|---|---|---|
| U12 | U12 | U12 | U12 | U12 | 156 |
| U12 | U12 | U12 | U12 | Lost | 6 |
| U12 | U12 | U12 | U12 | U2 | 1 |
| U12 | U12 | U12 | Lost | U12 | 2 |
| U12 | U12 | U12 | U2 | U12 | 3 |
| U12 | U12 | U12 | ambi | U12 | 4 |
| U12 | U12 | U12 | U12 | ambi | 1 |
| U12, ambi | U2 | U2 | U2 | U2 | 1 |
| U12, ambi | ambi | ambi | U12 | U12 | 1 |
| U12, ambi | U12 | U12 | U12 | U12 | 5 |

Table S3. Details of cases of losses and type conversions listed in Table S2. For loss cases the intron number refers to that of the one in the human orthologs.

| | **Type of change** | **Species** | **Gene** | **Intron number** | **Intron termini** | **Ensembl gene ID** |
|---|---|---|---|---|---|---|
| 1 | Loss | *T. nigroviridis* | *DCP2* (mRNA decapping enzyme 2) | 2 | AT-AC | GSTENG00007196001 (intronless) |
| 2 | Loss | *T. nigroviridis* | *C1orf164* (chromosome 1 open reading frame 164) | 9 | GT-AG | GSTENG00015184001 |
| 3 | Loss | *T. nigroviridis* | *XAB1* (XPA-binding protein 1) | 3 | GT-AG | GSTENG00003557001 |
| 4 | Loss | *T. nigroviridis* | *C21orf33* (chromosome 21 open reading frame 33) | 6 | AT-AC | GSTENG00027938001 |
| 5 | Loss | *T. nigroviridis* | *VAC14* (Vac14 homolog) | 8 | GT-AG | GSTENG00034358001 (intronless) |
| 6 | Loss | *T. nigroviridis* | *VAC14* (Vac14 homolog) | 16 | GT-AG | GSTENG00034358001 (intronless) |
| 7 | U12 -> U2 | *T. nigroviridis* | *EXOSC2* (Exosome complex exonuclease) | 5 | GT-AG | GSTENG00017201001 |
| 8 | Loss | *D. rerio* | *VPS16* (vacuolar protein sorting 16) | 9 | GT-AG | ENSDARG00000033588 |
| 9 | Loss | *D. rerio* | *VPS16* (vacuolar protein sorting 16) | 13 | GT-AG | ENSDARG00000033588 |
| 10 | U12 -> U2 | *D. rerio* | *DCTN6* (Dynactin subunit 6) | 3 | GT-AG | ENSDARG00000046084 |
| 11 | U12 -> U2 | *D. rerio* | *AOX1* (Aldehyde oxidase 1) | 2 | GT-AG | ENSDARG00000020054 |
| 12 | U12 -> U2 | *D. rerio* | *AOX1* (Aldehyde oxidase 1) | 3 | GT-AG | ENSDARG00000020054 |
| 13 | U12 -> U2 | D. rerio | *CSNK1G1* ( casein kinase 1, gamma 1) | 7 | GT-AG | ENSDARG00000033872 |

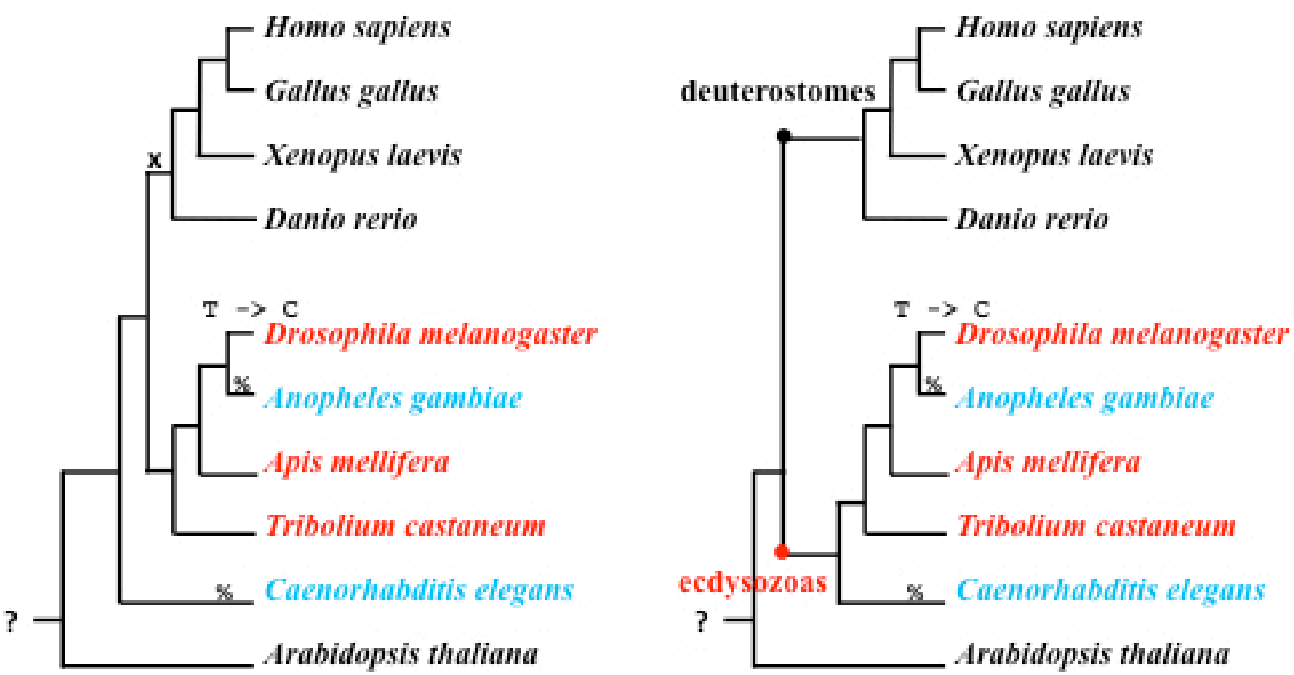
Homo sapiens
Gallus gallus
Xenopus laevis
Danio rerio
x
T -> C
Drosophila melanogaster
%
Anopheles gambiae
Apis mellifera
Tribolium castaneum
%
Caenorhabditis elegans
?
Arabidopsis thaliana
deuterostomes
Homo sapiens
Gallus gallus
Xenopus laevis
Danio rerio
T -> C
Drosophila melanogaster
%
Anopheles gambiae
Apis mellifera
Tribolium castaneum
ecdysozoas
%
Caenorhabditis elegans
?
Arabidopsis thaliana

Figure S1

<table>
<tr><th>Species</th><th></th><th>Intron 2</th><th></th><th>Intron 3</th></tr>
<tr><td>AOX1</td><td>type</td><td>5′ and 3′ junctions</td><td>type</td><td>5′ and 3′ junctions</td></tr>
<tr><td>H. sapiens</td><td>U12</td><td>AGGAAGAAGC|GTATCCTTT..GATCTTTAACTATACCTCTTCCAG|TTCGAC</td><td>U2</td><td>AGAGGATAAG|GTACCGTGC..GCTTTTCTTTTGCATTCTGAAG|GCATCA</td></tr>
<tr><td>M. musculus</td><td>U12</td><td>AGGAAGAATC|GTATCCTTT..TCTCTTTAACAGAGCTCCTTCCAG|TCCGAC</td><td>U2</td><td>AGGCGATCAG|GTAGGTGCA..GCTTCTCTTTTGCATTCTGAAG|GCATCA</td></tr>
<tr><td>R. norvegicus</td><td>U12</td><td>AGGAAGAACC|ATATCCTTT..TCTCTTTGACCGAGCTCCTTCCAG|TCCGAC</td><td>U2</td><td>AGAGCATCAG|GTGGGTGCA..GTTTCTCTTTTGCATTCTGAAG|GCATCA</td></tr>
<tr><td>C. familiaris</td><td>U12</td><td>AGGAAGAAGT|GTATCCTTT..TTTCCTTGACTCCCCTCCTTCCAG|TCTGTC</td><td>U2</td><td>CGAAGATAAG|GTATCCTAC..TCTTTGTGTTCCTCGTATGAAG|ACACTT</td></tr>
<tr><td>M. domestica</td><td>U12</td><td>AGGAAGAAAC|GTATCCTTT..TTTCTTTGACTACTGCCCTTCAAG|TCCATC</td><td>ambi</td><td>AAAAAATAAG|GTATCCTTC..ATTTCTTATTTTTGTTCTGAAG|ACATTA</td></tr>
<tr><td>G. gallus</td><td>U12</td><td>CGAAAGAGAC|GTATCCTTT..TTTTCCTTAATAGCGGAATTTCAG|TCCGTC</td><td>U12</td><td>AGAAGATACG|GTATCCTTA..TATTCACCTTAATGTTCCAAAG|ACACTA</td></tr>
<tr><td>D. rerio</td><td>U2</td><td>AGGAAAAAAT|GTATATTTT..GTATATGGATGTTTTTGTATGTAG|TGGGTT</td><td>U2</td><td>AGAGGATCAG|GTATTAGAG..AGGATTTTTTTTGTGTGTTTAG|TGATTT</td></tr>
<tr><td>T. nigroviridis</td><td>U12</td><td>AGAGACAGGC|GTATCCTTC..GCCTTTCCTTCACCGCCGCTTCAG|TGAGGC</td><td>U12</td><td>GGAGCATCAC|GTATCCTTC..GTTTGTCTTCACTCAGGACCAG|ACATTC</td></tr>
<tr><td>F. rubripes</td><td>U12</td><td>AGACAGAAAC|GTATCCTTC..GGCTTTCCTTCACTCTGACTTCAG|TGAGGC</td><td>U12</td><td>AAACCATCAC|GTATCCTTG..ACCGACCTTCACTCAGCATTAG|ACACCT</td></tr>
<tr><td>XDH</td><td>type</td><td>5′ and 3′ junctions</td><td>type</td><td>5′ and 3′ junctions</td></tr>
<tr><td>H. sapiens</td><td>U12</td><td>AGAAGAAAGT|GTATCCTGA..TCTCCTTAACTCTTGACCACCCAG|TGGGGC</td><td>U12</td><td>ACAAGATCGT|GTATCCTTT..TGACCTTAATCTGGGGTTCTAG|CCACTT</td></tr>
<tr><td>M. musculus</td><td>U12</td><td>AGAAGAAAGT|GTATCCTGA..TGTCCTTAACAAGAGGCTGCTCAG|TGGGGC</td><td>U12</td><td>ACAAGATCGT|GTATCCTTT..TGGCCTTAATCTGTGGTTCTAG|TCATTT</td></tr>
<tr><td>R. norvegicus</td><td>U12</td><td>AGAAGAAAGT|GTATCCTGA..TGTCCTTAACAAGTGGTTGTTCAG|TGGGGC</td><td>U12</td><td>ACAAGATTGT|GTATCCTTT..CAACCTTAATCTGTGGTTCTAG|TCATTT</td></tr>
<tr><td>C. familiaris</td><td>U12</td><td>AGAAGAAAAT|GTATCCTGA..ATTCCTTAACTCTCGACCATCCAG|TGCGGC</td><td>U12</td><td>ACAAGATCGT|GTATCCTTT..TGACCTTAATCCAGGGCCCCAG|CCACTT</td></tr>
<tr><td>M. domestica</td><td>U12</td><td>AGAAGAAAAT|GTATCCCTG..TTTTCCTTAACTCTTGCACTTTCA|GTGGGG</td><td>U12</td><td>AAAAAATTGT|GTATCCTTT..TATCCTTTACTTGAGTTTCCAG|CCACTT</td></tr>
<tr><td>G. gallus</td><td>ambi</td><td>CGAAGAAAAC|GTATCTGGT..TTTCCTTGATTCCTGTACTCTCAG|TGGGCC</td><td>U12</td><td>AGAAAATCCT|GTATCCTTT..TGACCTTAATCTATCATTTTAG|CCACCA</td></tr>
<tr><td>X. tropicalis</td><td>ambi</td><td>CGGAGAAAAT|GTACGGTTT..TTCCCTTAACCTCTTTCCATTCAG|TGGGAT</td><td>U12</td><td>ACAGAATACT|GTATCTTTT..CTTCTTAAACGCATAATTCCAG|ACACTA</td></tr>
<tr><td>D. rerio</td><td>U12</td><td>AGAAGAAGCT|GTATCCTTT..TTTCCTTAATGTTGTCACCTGCAG|TGGGTC</td><td>U12</td><td>ACCGGATTAT|GTATCTTTT..TATCCTTGATTCTCTCTTGAAG|TCACTA</td></tr>
<tr><td>F. rubripes</td><td>U2</td><td>AGGAGAAAAT|GTGAGCTCA..GTGTGTGTGTGTGTGTGTGTTCAG|TGGGAT</td><td>U12</td><td>AACAGCTACT|GTATCCTTC..TGGTCTTTAACTCCGGTTCCAG|TCACTA</td></tr>
<tr><td>T. nigroviridis</td><td>U2</td><td>AGGAGGAAAT|GTAAGCTCC..AACCATCTTTTCTTTAGCCTCCAG|TGAGGA</td><td>U12</td><td>AACAGCTGCT|GTATCCGTG..AGGTCTTTAACTGCGGTTTCAG|TCACTA</td></tr>
<tr><td>C. intestinalis</td><td>U2</td><td>AGAACAAAAC|GTGAGTGAC..ATTATGTGACTTTTTTTGTTTTAG|TTCGTT</td><td>U2</td><td>ACCGCATCGT|GTGAGTTTG..CGTTGTTTTTTTGTTTTTTCAG|ACATTT</td></tr>
<tr><td>A. mellifera</td><td>U12</td><td>AGAAATAAGT|GTATCCTTT..AAGTTTTCCTAACTAGAATAATAG|TACGTT</td><td>U12</td><td>GAATTATTAC|GTATCCTTT..TTTAATTTTTTAATTTAAATAG|ACATCT</td></tr>
<tr><td>A. gambiae</td><td>lost</td><td></td><td>lost</td><td></td></tr>
<tr><td>D. melanogaster</td><td>lost</td><td></td><td>lost</td><td></td></tr>
<tr><td>C. elegans</td><td>U2</td><td>AGAGATAAAT|GTGAGTTCT..TTTACGAGTTTATTTTATTTTAAG|TGAAGC</td><td>U2</td><td>GTGAAATCAA|GTGAGTTGG..TTTTAAAGCCTAATTGTTCCAG|ACATTT</td></tr>
</table>

Figure S2

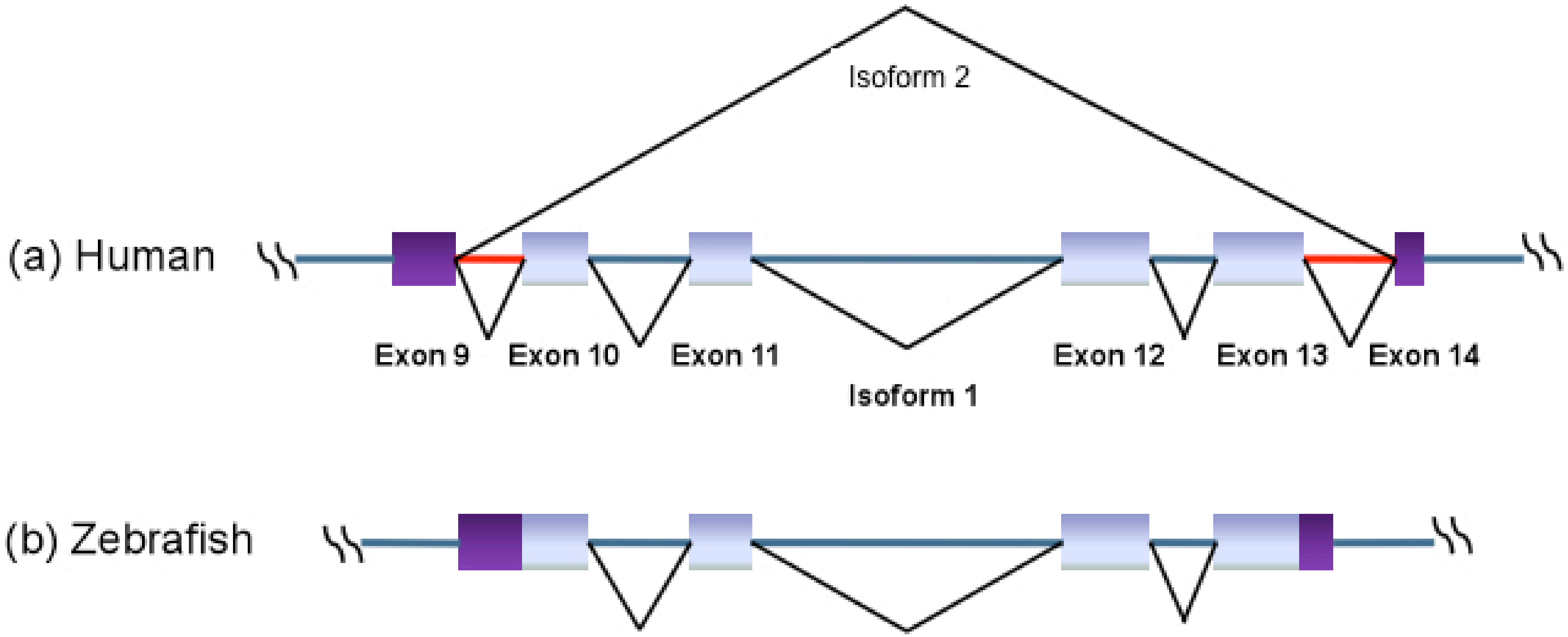
Isoform 2
(a) Human
Exon 9
Exon 10
Exon 11
Exon 12
Exon 13
Exon 14
Isoform 1
(b) Zebrafish

Figure S3

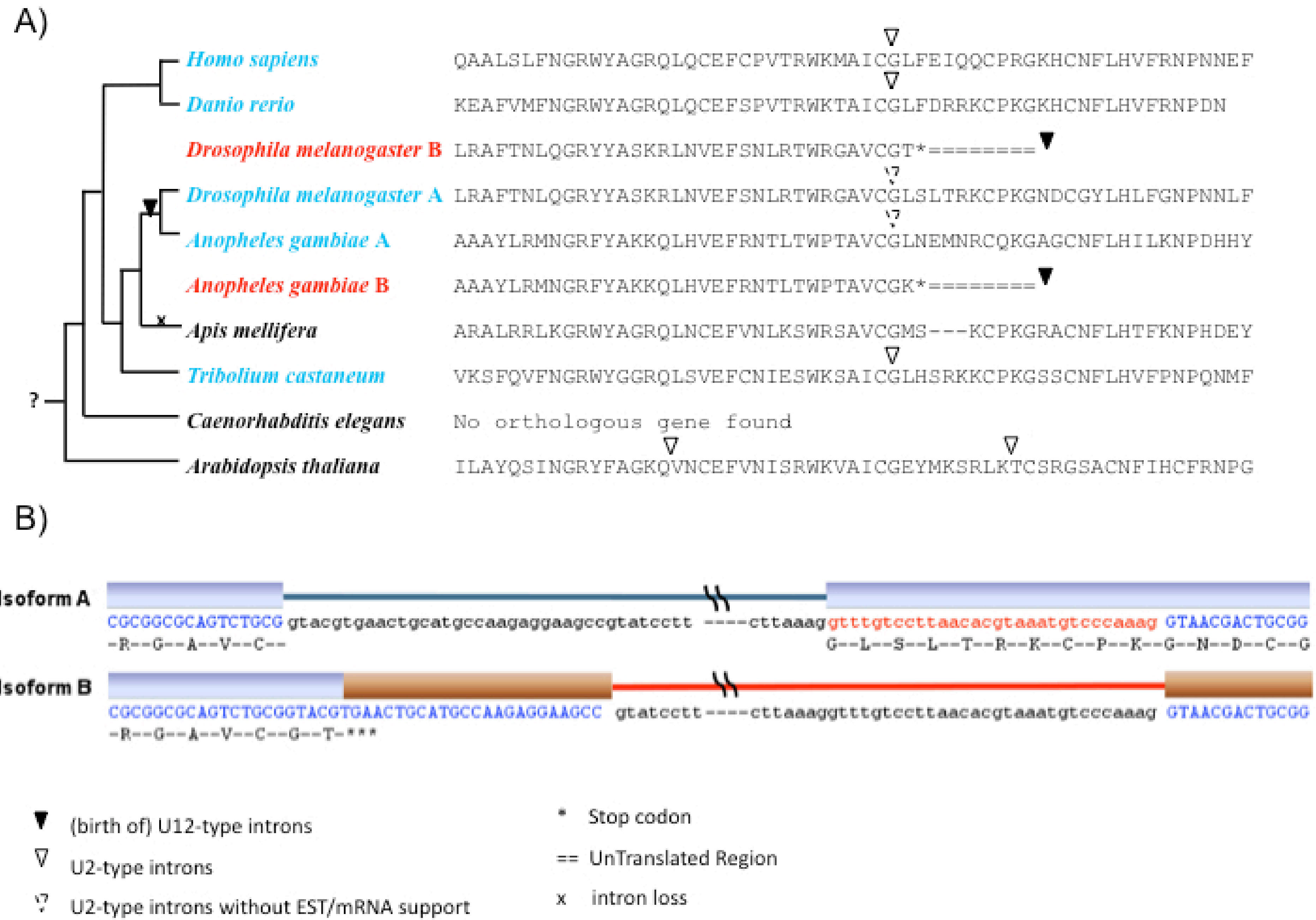
A)
Homo sapiens
QAALSLFNGRWYAGRQLQCEFCPVTRWKMAICGLFEIQQCPRGKHCNFLHVFRNPNNEF
Danio rerio
KEAFVMFNGRWYAGRQLQCEFSPVTRWKTAICGLFDRRKCPKGKHCNFLHVFRNPDN
Drosophila melanogaster B
LRAFTNLQGRYYASKRLNVEFSNLRTWRGAVCGT*========
Drosophila melanogaster A
LRAFTNLQGRYYASKRLNVEFSNLRTWRGAVCGLSLTRKCPKGNDCGYLHLFGNPNNLF
Anopheles gambiae A
AAAYLRMNGRFYAKKQLHVEFRNTLTWPTAVCGLNEMNRCQKGAGCNFLHILKNPDHHY
Anopheles gambiae B
AAAYLRMNGRFYAKKQLHVEFRNTLTWPTAVCGK*========
Apis mellifera
ARALRRLKGRWYAGRQLNCEFVNLKSWRSAVCGMS---KCPKGRACNFLHTFKNPHDEY
Tribolium castaneum
VKSFQVFNGRWYGGRQLSVEFCNIESWKSAICGLHSRKKCPKGSSCNFLHVFPNPQNMF
Caenorhabditis elegans
No orthologous gene found
Arabidopsis thaliana
ILAYQSINGRYFAGKQVNCEFVNISRWKVAICGEYMKSRLKTCSRGSACNFIHCFRNPG
?
x
B)
Isoform A
CGCGGCGCAGTCTGCG gtacgtgaactgcatgccaagaggaagccgtatcctt ----cttaaag gtttgtccttaacacgtaaatgtcccaaag GTAACGACTGCGG
-R--G--A--V--C--
G--L--S--L--T--R--K--C--P--K--G--N--D--C--G
Isoform B
CGCGGCGCAGTCTGCGGTACGTGAACTGCATGCCAAGAGGAAGCC gtatcctt----cttaaaggtttgtccttaacacgtaaatgtcccaaag GTAACGACTGCGG
-R--G--A--V--C--G--T-***
(birth of) U12-type introns
U2-type introns
U2-type introns without EST/mRNA support
* Stop codon
== UnTranslated Region
x intron loss

Figure S4
